# Methodological refinement of Aldara-induced psoriasiform dermatitis model in mice

**DOI:** 10.1038/s41598-019-39903-x

**Published:** 2019-03-06

**Authors:** Szabina Horváth, Rita Komlódi, Anikó Perkecz, Erika Pintér, Rolland Gyulai, Ágnes Kemény

**Affiliations:** 1 Department of Dermatology, Venereology and Oncodermatology, University of Pécs, H-7632 Pécs, Akác str. 1, Hungary; 2 Department of Pharmacology and Pharmacotherapy, University of Pécs, Medical School, H-7624 Pécs, Szigeti str. 12, Hungary; 3 János Szentágothai Research Center, University of Pécs, H-7624 Pécs, Ifjúság str. 20, Hungary; 4 Department of Medical Biology, University of Pécs, Medical School, H-7624 Pécs, Szigeti str. 12, Hungary

## Abstract

Imiquimod (IMQ)-induced skin inflammation is currently the most widely accepted psoriasis animal model, however, it features several limitations. We have modified the IMQ-model to minimize its systemic effects towards effectively maintaining the characteristic skin reactions. The original protocol (OP) uses 62.5 mg Aldara cream (or vaseline) on the shaved back skin of mice for 4 days. In contrast, in our modified protocol (MP) 25 mg Aldara and vaseline are applied simultaneously in separate Finn chambers over the dorsal skin of mice. In both the OP and MP groups, histology showed unequivocal hallmarks of psoriasiform dermatitis. Additionally, skin scaling and blood perfusion values were similar. While Aldara elicited significantly increased skin thickness in the MP group, significant weight loss, spleen enlargement, increased inflammatory cytokine levels in plasma, and treatment related death were only observed in the OP group. Our new method reproduces psoriatic skin alterations highlighting considerably reduced systemic inflammatory reactions. Possessing psoriasiform and control skin areas on the same mouse also reduces inter-individual differences. Additionally, the new method permits prolonged IMQ treatment studies to mimic the chronic nature of psoriasis. Finally, our experimental approach may also be used in other mouse models, to prevent the undesired systemic effects of topically applied drugs.

## Introduction

Aldara (5% imiquimod)-induced acute skin inflammation became the most widely used animal model of psoriasis since it was first published in 2009^1^. In this model, 62.5 mg Aldara is smeared onto the shaved dorsal skin of mice for 5 or 6 days to induce scaly skin lesions resembling plaque type psoriasis. While the model does not exactly recapitulate human psoriasis^2^, imiquimod treatment of mouse skin can exert specific cytokine expression patterns, histopathological alterations and cellular infiltrates similar to what is observed in psoriatic patients^3^. Imiquimod is able to activate proinflammatory signaling pathways via the ligation of TLR7/8 upon dermal dendritic cells (DDCs), however, it is likely that other mechanisms such as NLRP1 inflammasome activation (pyroptosis)^4^, direct activation of TRPA1 non-selective cation channels located on immune cells and peripheral nerve endings^5^ also contributes to its proinflammatory effects. While it is generally accepted that IL-17 producing Th17 cells are the main cell type responsible for the development of the skin inflammation, other cells, such as subsets of the γδT17 cells also contribute to the immune reaction^6^.

Aldara-induced skin inflammation has several advantages compared to previous models of psoriasis, including rapid and reproducible skin response. Animals do not need pathogen free conditions, as seen in xenotransplantation models, and it is relatively inexpensive^7^. On the other hand, this model possesses several limitations. Among these, the overuse of the Aldara cream and its ingestion is the most problematic since it can cause severe systemic inflammation indicated by splenomegaly, worsened general condition and untimely death of the animals^8^. This can contribute to the phenotypic variability observed in this model and it may likely be the reason for the different treatment regimens applied by various authors^1,9,10^.

Therefore, our aim was to refine the Aldara-induced psoriasiform inflammatory model to determine the optimal dose of cream with maintained pathological alterations present in psoriasis and to eliminate systemic effects by reducing the possibility of ingestion and direct scratching of the treated dorsal skin.

## Results

### Clinical signs of Aldara-induced skin inflammation

**Figure 1:**
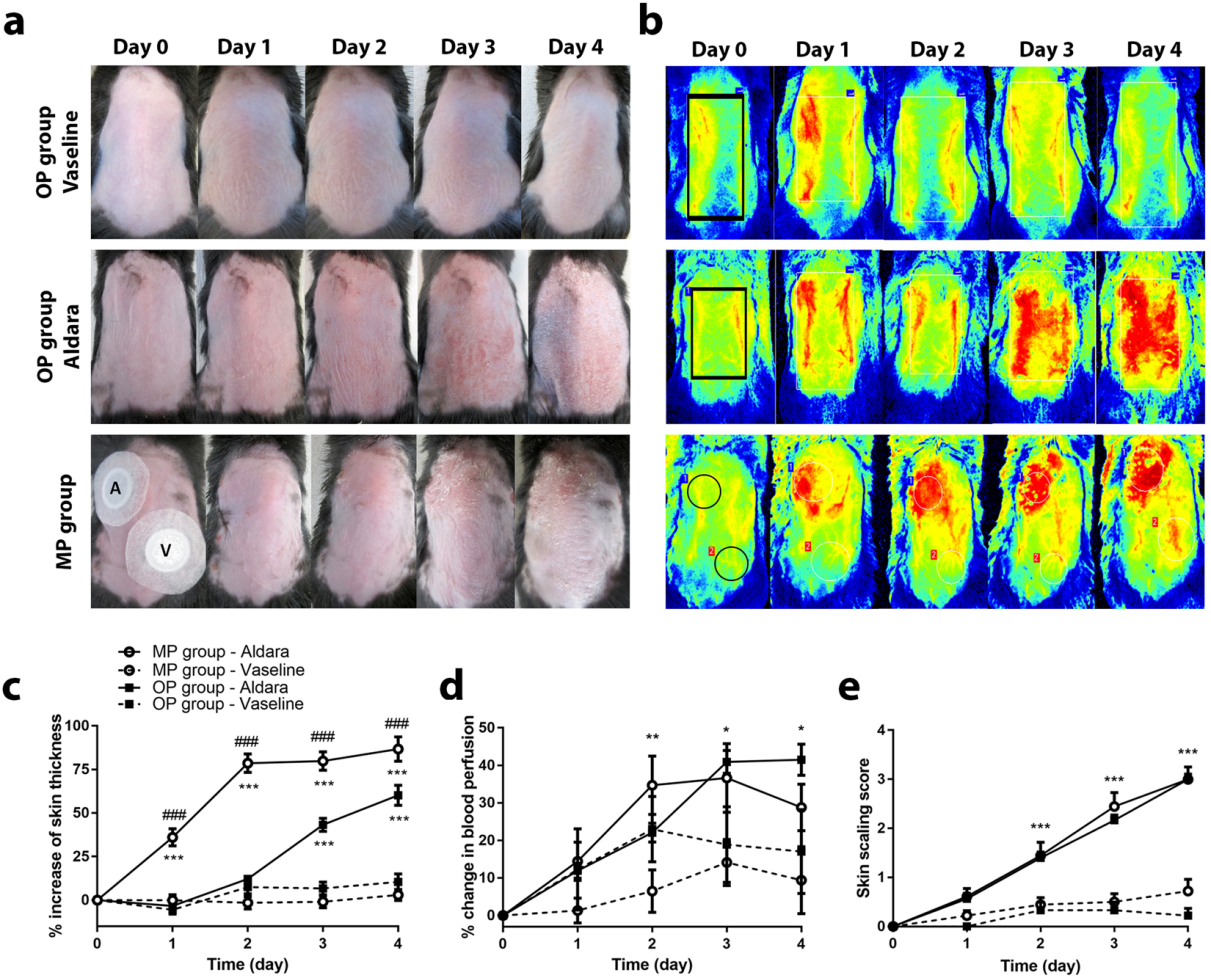
Comparison of functional parameters in Aldara-induced psoriasiform dermatitis using original and modified protocols. (**a**) Clinical signs of topical Aldara treatment on C57BL/6 mice throughout the 5 day experiment. 62.5 mg vaseline (OP group - Vaseline) or Aldara (OP group - Aldara) was applied to the back skin of mice. 25–25 mg vaseline (V) or Aldara (A) was applied to the shaved back skin of animals using Finn chambers (MP group). (**b**) Representative images of blood perfusion changes on C57BL/6 mice dorsal skin induced by topical application of vaseline or Aldara in the OP group and in the MP group using Finn chambers. (**c**) Percent increase of back skin thickness after vaseline or Aldara treatment in OP or MP groups compared to day 0 baseline values. Data are mean ± SEM for n = 15/group. ***p < 0.001 vaseline vs. Aldara-treated sites, ^###^p < 0.001 Aldara-treated OP group vs. Aldara-treated MP group, based on repeated measures 2-way ANOVA followed by Bonferroni’s post hoc test. (**d**) Percent change of blood perfusion in dorsal skin after vaseline or Aldara treatment compared to the day 0 values. Data are mean ± SEM for n = 15/group. *p < 0.05; **p < 0.01 vaseline vs. Aldara-treated sites, based on repeated measures 2-way ANOVA followed by Bonferroni’s post hoc test. (**e**) Skin scaling scores after vaseline of Aldara treatment in OP group or MP group. Scaling was graded from 0 to 4 (0: absent to 4: severe). Data are mean ± SEM for n = 7–15/group. ***p < 0.001 vaseline vs. Aldara-treated sites, based on 2-way ANOVA followed by Bonferroni’s post hoc test. OP: original protocol, MP: modified protocol.

Clinical signs of psoriasis, such as skin thickening, erythema, and scaling were consistently observed in Aldara treated animals, however, they were not seen in vaseline-treated skin using the two different disease induction techniques in C57BL/6 mice (Fig. 1a). Erythema developed following the second treatment using Aldara, and soon thereafter, scaling appeared on the third day which continually increased in severity up to the end of the experiment in both OP and MP group (Fig. 1a).

### Similar blood perfusion results in OP and MP treatment groups

Aldara treatment induced elevated microcirculation in the dorsal skin compared to the vaseline-treated sites, represented by the red areas on the color pictures assessed by LASCA laser Speckle instrument (Fig. 1b). Significant increase in dorsal skin blood flow was observed using both Aldara treatment techniques compared to the control group or skin area, reaching maximal responses on day 3. More pronounced differences were measured between vaseline- or Aldara-treated sites in MP group on day 2 and 3. The same percent change values are noted in the Aldara-induced microcirculation in both OP and MP groups (Fig. 1d).

### Aldara treatment induced significantly higher back skin thickness in the MP group

Application of Aldara cream significantly increased the thickness of dorsal skin compared to vaseline treated control mice in the OP group from day 3, while in the MP group a significant difference was observed following the first treatment. Aldara (IMQ) generated skin edema/infiltration in both the OP group and MP group, resulted in a constant increase until the end of the experiment, ultimately attaining a maximum value of 60% or 86% in OP or MP group, respectively. Dorsal skin swelling/infiltration was significantly higher in the Aldara-treated MP group when compared to the OP group on each day of experiment (Fig. 1c).

### No significant difference was observed in skin scaling of OP and MP treatment groups

Skin scaling was formed only on the Aldara-treated dorsal skin of the MP group, but not on the vaseline-treated skin areas. Increasing skin scaling scores were observed throughout the 5-day experiment in the Aldara-treated site and there was no significant difference between the OP and MP groups (Fig. 1e).

### Histological analysis of Aldara-induced dermatitis in OP or MP groups

**Figure 2:**
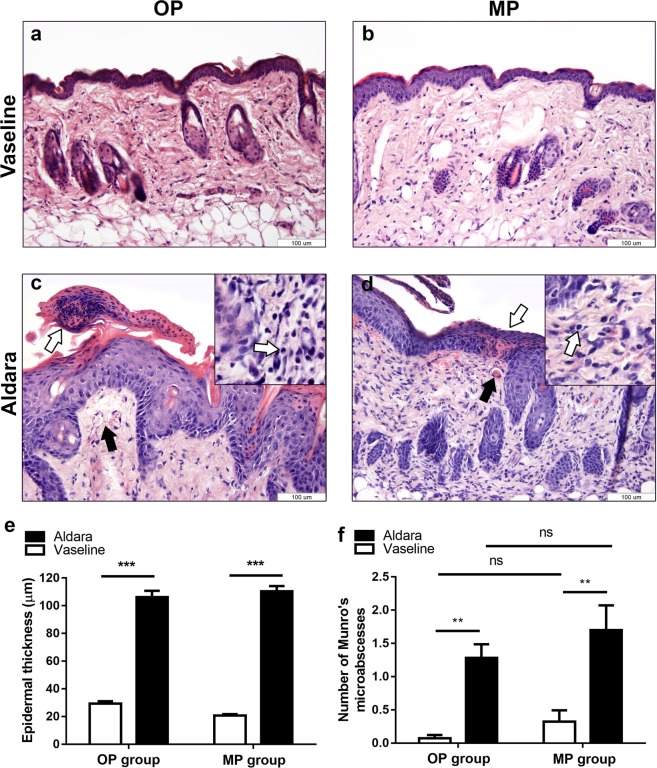
Comparison of histological alterations following Aldara- or vaseline-treatment on the dorsal skin of C57BL/6 mice using OP or MP. Vaseline-treated control skin of OP group (**a**) or MP group (**b**) at 200x magnification. Aldara-treated skin of OP group (**c**) or MP group (**d**) at 200x magnification. Open arrow: Munro’s microabscesses, black arrow: dilated capillaries. White arrows in the inserts indicate T-cell infiltration in the dermis. Thickness of epidermal layer (**e**) and number of Munro’s microabscesses (**f**) throughout the sections after different treatments. Hematoxylin-eosin staining. Data are mean ± SEM for n = 5/group. **p < 0.01 ***p < 0.001 vaseline vs. Aldara-treated sites, based on one-way ANOVA followed by Bonferroni’s post hoc test. OP: original protocol, MP: modified protocol.

Histological sections of Aldara-treated skin obtained from the “whole back skin” model or “Finn chamber” model clearly showed typical alterations present in psoriasis (keratinocytes hyperproliferation, hyperkeratosis, parakeratosis, Munro microabscesses) in both experimental groups (Fig. 2). Accumulation of neutrofils in the epidermis produced several Munro microabscesses following Aldara treatment (Fig. 2c,d open arrows). Tortured, dilated capillaries appeared in the dermal region of skin samples in both experimental paradigms (Fig. 2c,d black arrows). T-cell infiltration was also observable in the dermis of Aldara-treated skin samples of OP or MP groups (Fig. 2c,d insert, white arrows). Histopathological scoring assessing characteristic parameters in psoriasis (Munro’s microabscesses and thickness of epidermal layer) was not significant between Aldara-treated skin samples of OP group when compared to the MP group (Fig. 2e,f).

### Similar Ki-67 and CD11b expression pattern in OP or MP groups

**Figure 3:**
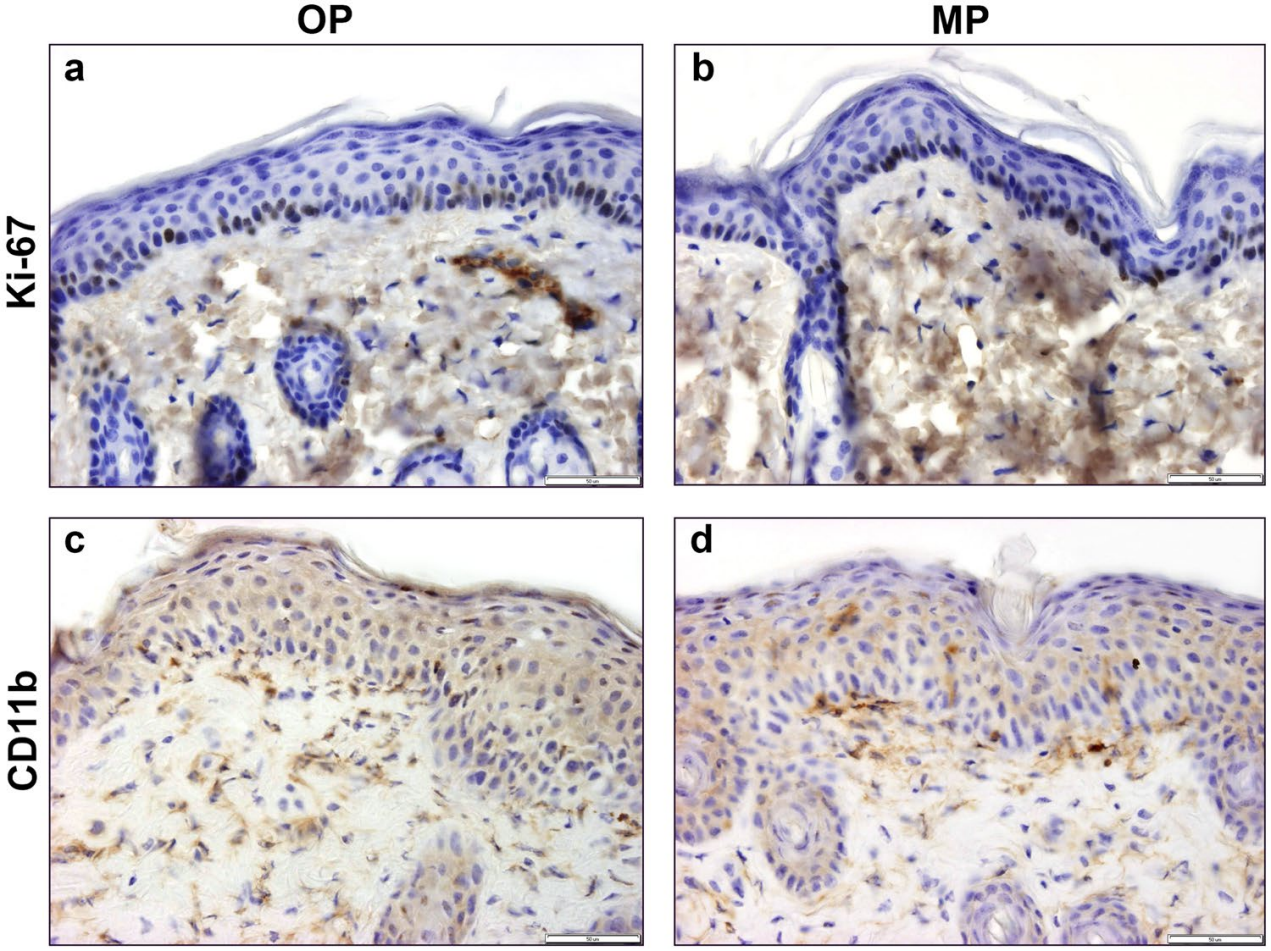
Comparison of Ki-67 or CD11b immunoreactivity of Aldara-treated dorsal skin samples of C57BL/6 mice using OP or MP. Nuclear proliferation marker Ki-67 immunostaining on OP (**a**) and MP (**b**) dorsal skin tissue samples at 400x magnification. Dermal dendritic cells were labelled with anti-CD11b antibody on OP (**c**) and MP samples (**d**) at 400x magnification.

Distribution of Ki-67+ cells in the stratum basale of Aldara-treated skin samples of OP or MP groups indicate similar cell proliferation rate induced by the conventional or the modified methods (Fig. 3a,b). CD11b+ dermal dendritic cells were present in higher number following Aldara treatment in both experimental groups (Fig. 3c,d).

### Reduced systemic inflammatory response in the MP group

**Figure 4:**
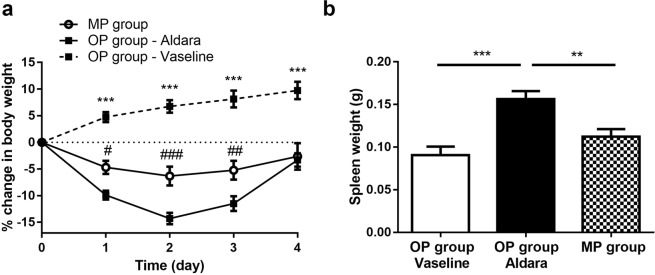
Modified protocol reduced the systemic effect of IMQ treatment in C57BL/6 mice. (**a**) Percent change of body weight after vaseline or Aldara treatment using two different methods. Data were expressed as percent change of body weight compared with day 0 baseline initial values. Data are mean ± SEM for n = 15/group. ^#^p < 0.05; ^##^p < 0.01; ^###^p < 0.001 Aldara-treated OP group vs. Aldara-treated MP group, ***p < 0.001 vaseline vs. Aldara-treated group, based on 2-way ANOVA followed by Bonferroni’s post hoc test. (**b**) Effect of the two different disease induction techniques for the development of the splenomegaly. Spleens were collected and weighted at the end of the experiment. Data are mean ± SEM for n = 15/group. **p < 0.01; ***p < 0.001, based on one-way ANOVA followed by Bonferroni’s post hoc test. OP: original protocol, MP: modified protocol.

An elevation in the percentile change of body weight was detected in the vaseline-treated control OP group while significant weight loss was registered throughout the 5-day experiment in the Aldara-treated OP and MP groups. Body weight loss was more remarkable in the “whole back” model group compared to the “Finn chamber” model group after the first, second and third Aldara treatments (Fig. 4a). Spleen weights were at or about 0.09 gram in the vaseline-treated OP group at the end of the experiment and we found significantly enlarged spleen tissues following the fourth day of Aldara treatment in the OP group (0.16 gram). The average spleen tissue weight in the MP group (“Finn chamber” model) was similar to the vaseline-treated OP group (0.11 gram) (Fig. 4b).

### Plasma concentration of inflammatory cytokines is only increased in the OP group

**Figure 5:**
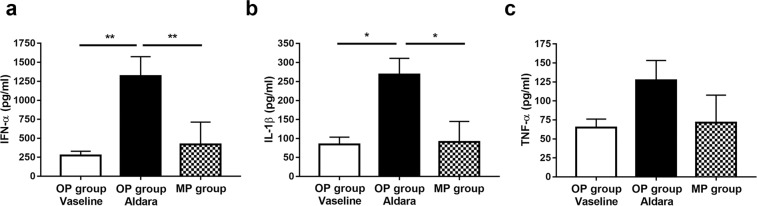
MP method diminished the production of inflammatory cytokines in plasma samples. (**a**) IFN-α, (**b**) IL-1β, and (**c**) TNF-α cytokine concentration 6 hrs after the first IMQ treatment in the plasma samples of C57BL/6 mice. Data are mean ± SEM for n = 5/group. *p < 0.05; **p < 0.01, based on one-way ANOVA followed by Bonferroni’s post hoc test. OP: original protocol, MP: modified protocol.

IL-1β and IFN-α cytokine concentrations were increased (p < 0.05) in the peripheral blood samples of mice in the OP group 6 hrs following the first Aldara treatment compared to the vehicle-treated group. Aldara did not enhance the levels of these cytokines in the MP group. TNF-α concentration was elevated in the case of Aldara-treated OP group compared to the MP group following the first treatment, however, significant differences were not detected between the Aldara-treated and the vaseline-treated control groups (Fig. 5).

## Discussion

Since its first introduction in 2009, the IMQ-induced psoriasis mice model has become the most widely used animal study in the field of psoriasis. In their recent review, Hawkes *et al*. relates to the exponentially increasing number of IMQ-model papers as the ‘Snowballing Literature on Imiquimod-Induced Skin Inflammation in Mice’^7^. The popularity of the method is likely associated with several advantages it offers to scientists: it is easy to use, provides quick results, it is versatile (can be combined with other methods, such as genetically modified animals), and it is relatively inexpensive. However, the method has several drawbacks, which require special attention and consideration when interpreting scientific data. Among these, as noted in the paper, Hawkes *et al*., first lists the ‘unintended consequences of topical treatment’, which highlights systemic side effects of imiquimod application. In their original paper, van der Fits *et al*. describes a significant spleen enlargement with an approximately 2-fold weight increase in the animals following 5 to 6 days of IMQ treatment^1^. Indeed, in most publications, IMQ-treated animals develop splenomegaly during the experimental procedure, clearly demonstrating how topical treatment of mice with IMQ results in systemic effects^8,11,12^. Moreover, topical IMQ treatment in mice leads to robust systemic inflammation (marked by elevated inflammatory cytokine levels in blood) and severe dehydration (often requiring the administration of subcutaneous fluids). Although this phenomenon is frequently overlooked or disregarded when interpreting data generated by the imiquimod model, systemic disease may significantly influence the results. Dehydration, for example, causes significant changes in skin structure, leading to thinner, tighter and flakier skin. Systemic and cutaneous dehydration may also severely impact skin barrier and immune functions, and thus, may substantially influence the outcome of the experiments. Since in the traditional model IMQ-treated animals will develop these symptoms to varying extent, while the control mice will not, resulting in a very high likelihood for inherent experimental bias.

Systemic symptoms are likely associated with at least two factors. First, in mice, the area of treated skin in the traditional model is considerably large, approximately 15% of the total body surface area^13^. Imiquimod treatment over such a large skin area possibly leads to general symptoms, and is also observed in humans^14,15^. Secondly, the grooming behavior of the animals results in the ingestion of imiquimod, generating type I IFN induction and activation in the gut, and consequently, leading to systemic responses^8^. This can be more problematic if control and treated animals are co-housed, as in this case, control animals may also ingest a significant amount of imiquimod from their littermates. Moreover, once ingested, imiquimod tends to modify the gut microbiome, which can have substantial consequences regarding (skin) immune function^16–18^. While ingestion of imiquimod can be avoided by housing the animals separately, and with the use of Elizabethan collars to prevent self-licking, the large treated surface area remains a potential experimental pitfall.

In reference to our research, we are introducing a new method for the induction of psoriasiform dermatitis in mice using Finn chambers. This new technique proved to be sufficient to elicit skin reactions such as edema, infiltration, scaling, increased blood perfusion, and psoriasiform histopathological alterations, similar to the classical imiquimod model. However, our new method leads to considerably reduced systemic inflammatory reactions in the animals, as indicated by the moderate splenomegaly and weight loss, as well as little or no significant increase of IL-1β, TNF-α and IFN-α concentrations in the blood of the animals. We assume the decreased systemic response is due both to the reduced IMQ-treated skin area and to the prevention of oral intake. A further advantage of the method is the psoriasiform and the control skin areas are on the same mice. This fact decreases the likelihood of inter-animal differences and ensures the model is even more cost-effective. On the other hand, one can argue, in consideration of this method, imiquimod may elicit some systemic effects, which may theoretically influence the control data. If this is a potential concern for the experimental design, we suggest using separate animals as controls.

Distinctively, the animals used in the experiments did not show any sign of significant systemic disease, therefore, our novel method is ideally suitable to perform prolonged imiquimod treatment studies. Thus, it may potentially contribute to the refinement of the IMQ model, to more accurately mimic the chronic nature of psoriasis, and also prove beneficial in further studies of psoriasis comorbidities.

Finally, our experimental approach may also be used in mouse model experiments with other topically applied drugs (such as TPA and acetone), to prevent the ingestion and the systemic consequences of these compounds.

In conclusion, our new method of using Finn chambers can be used to elicit the IMQ-induced psoriasiform dermatitis in mice without the systemic effects associated with the traditional technique.

## Materials and Methods

### Animals

Experiments were performed on female C57BL/6 mice (8–10 weeks, 20–25 g). The animals were bred and kept under standard pathogen-free conditions in the Laboratory Animal House of the Department of Pharmacology and Pharmacotherapy of the University of Pécs, at 24–25 °C, provided with food and water *ad libitum*. All procedures were carried out according to the 1998/XXVIII Act of the Hungarian Parliament on Animal Protection and Consideration Decree of Scientific Procedures of Animal Experiments (243/1988). All experiments were approved by the Ethics Committee on Animal Research of the University of Pécs, in full accordance to the Ethical Codex of Animal Experiments, and a license was assigned (license number: BA 02/2000-36/2017).

### Induction of psoriasiform skin inflammation

The dorsal skin of mice was shaved using an electric shaver, and the remaining hairs were completely removed with depilatory cream one day prior to the first Aldara treatment. Two experimental paradigms were used for induction of psoriasiform dermatitis. In the original protocol (OP) group, 62.5 mg Aldara cream (5% IMQ) was applied on the shaved back skin of C57BL/6 mice on each day of the 4-day experiment^1^. In control animals, vaseline was used and in the same amount. In the modified protocol (MP) group, two Finn chambers (8 mm FinnChambers on Scanpor, SmartPractice, USA) were placed on the dorsal skin of each mouse, one filled with 25 mg Aldara, the other with 25 mg vaseline. Finn chambers were applied on the dorsal skin of mice for 8 hrs which proved to be sufficient for the complete penetration of the creams into the skin. Treatments were repeated over the next four days ensuring the same areas were treated daily. All procedures were done under ketamine (100 mg/kg i.p., Richter, Hungary) and xylazine (5 mg/kg i.p., Lavet, Hungary) anesthesia and at the end of the experiments, the anesthetized animals were sacrificed using cervical dislocation. Treated dorsal skin, spleen tissue and blood samples were all collected for further analysis.

### Measurement of dorsal skin thickness

Double-fold dorsal skin thickness was measured and averaged at two distinct sites of the OP group, while the 8 mm treated areas were measured in the MP group using an engineer’s micrometer (Moore and Wright, Sheffield, England) with 0.1 mm accuracy, prior to treatment with Aldara or control cream on each day of the experiments. Data were expressed as percent increase of back skin thickness compared with the initial values.

### Measurement of blood perfusion changes

Blood perfusion was detected with Laser Speckle Contrast Analysis (LASCA) laser Doppler (Perimed, Sweden) method prior to the application of Aldara and vaseline on each day of the experiment. Measurements from this instrument are displayed as arbitrary perfusion units. Regions of interest (ROI) were selected according to the treated area on the dorsal skin (Aldara/vaseline treated whole back of OP group or the area covered by the Finn chambers in MP group) and mean perfusion was generated by PimSoft software (Perimed, Sweden). Data were expressed as percentage of blood perfusion change compared to the initial values.

### Skin scaling score

The Aldara- or vaseline-treated dorsal skin area of C57BL/6 mice were evaluated daily by three trained dermatologists who were blinded for both experimental protocols. Skin scaling was scored, ranging from 0 to 4, as represented in the following: 0 – none, 1 – slight, 2 – moderate, 3 – marked, 4 – maximum.

### Histology

Excised dorsal skin tissue samples were formalin-fixed (6%) and embedded in paraffin, and 5 µm sections were cut and stained with hematoxylin-eosin for further histological analysis. Scoring parameters and values were determined on the basis of the signs characteristic to psoriasis at 200x magnification: (1) thickness of skin epidermal layer measured by AnalySIS software (2) Number of Munro’s microabscesses throughout the section.

### Immunohistochemistry

5 µm snap frozen tissue samples were sectioned by cryostat microtome. Endogenous peroxidase activity was blocked by incubation with 0.3% hydrogen peroxide for 15 min. Nonspecific secondary antibody binding was inhibited using BSA for 20 min. Tissue sections were incubated with rabbit polyclonal anti-mouse Ki-67 (AB9260, EMD Millipore Corporation, Temecula, CA, USA; 1:250), or rabbit polyclonal anti-mouse CD11b (NB110-89474, Novus Biologicals, CO 80112, USA; dilution 1:400) primary antibodies for 1 hour at room temperature. This was followed by the incubation with horseradish peroxidase (HRP) conjugated secondary antibody for 30 min. Finally, visualization of target proteins was performed by diaminobenzidine development and nuclear staining with hematoxylin.

### Measurement of proinflammatory cytokines

The concentration of proinflammatory cytokine TNF-α, IL-1β and IFN-α was measured from the peripheral blood samples collected 6 hours following the first Aldara treatment by ELISA method (Mouse TNF-α or IL-1β ELISA Set, catalogue number: 555268 or 559603, respectively, BD Biosciences Eastern Europe, Heidelberg, Germany; Mouse IFN-α Platinum ELISA, catalogue number: BMS6027, Invitrogen, USA). The principal of the assay is to coat capture antibodies specific for the protein of interest onto the wells of a microplate. Standards and unknown samples are added into the plate. During the first incubation step, the antigen binds to the capture antibody. Following incubation, unbound proteins are removed by washing and a biotinylated detection antibody is added to the wells to form capture antibody-antigen-detection antibody complex. After the removal of unbound detection antibodies, streptavidin-HRP conjugate is added and binds to the biotinylated detection antibody. Following the last incubation and washing cycle to remove the unbound HRP conjugate, a substrate solution is added and is converted by the HRP enzyme to a detectable color product read by spectrophotometry. The color intensity is directly proportional to the concentration of antigen present in the unknown sample.

### Statistical analysis

Results are expressed as mean ± S.E.M. Comparisons between Aldara- or vaseline-treated OP group and MP group were performed using a two-way ANOVA, followed by Bonferroni’s *post hoc* test for skin thickness, blood flow perfusion, skin scaling, and body weight. Splenomegaly, cytokine concentration and histology score data were evaluated by one-way ANOVA, followed by Bonferroni’s *post hoc* test. Statistical analysis was done using GraphPad Prism 5 for Windows (GraphPad Software, USA). Probability values p < 0.05 were regarded as significant.

## Data Availability

The datasets generated during and/or analysed during the current study are available from the corresponding author on reasonable request.

## References

[1] van der Fits L (2009). Imiquimod-induced psoriasis-like skin inflammation in mice is mediated via the IL-23/IL-17 axis. J. Immunol..

[2] Swindell WR (2017). Imiquimod has strain-dependent effects in mice and does not uniquely model human psoriasis. Genome Med..

[3] Yoshiki R (2014). IL-23 from Langerhans Cells Is Required for the Development of Imiquimod-Induced Psoriasis-Like Dermatitis by Induction of IL-17A-Producing γδ T Cells. J. Invest. Dermatol..

[4] Walter A (2013). Aldara activates TLR7-independent immune defence. Nat. Commun..

[5] Kemény, Á. *et al*. TRPA1 acts in a protective manner in imiquimod-induced psoriasiform dermatitis in mice. *J. Invest. Dermatol*. 10.1016/j.jid.2018.02.040 (2018). 10.1016/j.jid.2018.02.040 29550417

[6] Gray EE (2013). Deficiency in IL-17-committed Vγ4 + γδ T cells in a spontaneous Sox13-mutant CD45.1+ congenic mouse substrain provides protection from dermatitis. Nat. Immunol..

[7] Hawkes JE, Gudjonsson JE, Ward NL (2017). The Snowballing Literature on Imiquimod-Induced Skin Inflammation in Mice: A Critical Appraisal. J. Invest. Dermatol..

[8] Grine L (2016). Topical imiquimod yields systemic effects due to unintended oral uptake. Sci. Rep..

[9] Kim HR (2014). Reactive oxygen species prevent imiquimod-induced psoriatic dermatitis through enhancing regulatory T cell function. PLoS One.

[10] Vinter, H. *et al*. TNFα plays a significant role in the Aldara-induced skin inflammation in mice. *Br. J. Dermatol*. n/a-n/a 10.1111/bjd.14320 (2015).

[11] Madsen M (2018). Imiquimod-Induced Psoriasis-Like Skin Lesions Do Not Accelerate Atherosclerosis in Low-Density Lipoprotein Receptor-Deficient Mice. Am. J. Pathol..

[12] Kuang Y-H (2018). Topical Sunitinib ointment alleviates Psoriasis-like inflammation by inhibiting the proliferation and apoptosis of keratinocytes. Eur. J. Pharmacol..

[13] Cheung MC (2009). Body Surface Area Prediction in Normal, Hypermuscular, and Obese Mice. J. Surg. Res..

[14] Pachman DR (2012). Randomized clinical trial of imiquimod: an adjunct to treating cervical dysplasia. Am. J. Obstet. Gynecol..

[15] Adams S (2012). Topical TLR7 agonist imiquimod can induce immune-mediated rejection of skin metastases in patients with breast cancer. Clin. Cancer Res..

[16] Vetizou M (2015). Anticancer immunotherapy by CTLA-4 blockade relies on the gut microbiota. Science.

[17] Zakostelska Z (2016). Intestinal Microbiota Promotes Psoriasis-Like Skin Inflammation by Enhancing Th17 Response. PLoS One.

[18] Zanvit P (2015). Antibiotics in neonatal life increase murine susceptibility to experimental psoriasis. Nat. Commun..

